# “You Cannot Be Yourself”: Identity disruption, stigma, and the lived experience of anal fistula

**DOI:** 10.1371/journal.pone.0345581

**Published:** 2026-03-23

**Authors:** Karin Adamo, Fredrik Brännström, Jeaneth Johansson, Karin Strigård

**Affiliations:** 1 Department of Diagnostics and Intervention, Umeå University, Sweden; 2 Department of Diagnostics and Intervention, Umeå University, Sweden and Department of Surgery, St Göran Hospital, Stockholm, Sweden; 3 Luleå University of Technology, Department of Social Sciences Technology and Art, Sweden; 4 Halmstad University, Business, Innovation and Sustainability, Sweden; 5 Department of Diagnostics and Intervention, Umeå University, Sweden; Shuguang Hospital, CHINA

## Abstract

Anal fistula is a complex and often prolonged condition that significantly impacts patients’ daily lives and psychological well-being. This qualitative study explored how individuals living with anal fistula experience stigma, disruption, and identity strain in everyday life. Fifteen participants undergoing active treatment were interviewed at two hospitals. Data was analyzed using qualitative content analysis. Findings show that living with anal fistula was marked by shame, uncertainty, and social withdrawal, often contributing to an altered or fractured sense of self. Participants navigated this experience through three identity-shaping mechanisms: Knowledge Uncertainty, Expectations and Experiences, and Quality-of-Life. These mechanisms influenced how participants made sense of their condition, coped with invisibility, and negotiated bodily control in the context of pain and stigma. While many struggled to maintain a coherent identity in the face of chronic symptoms, interactions with empathetic healthcare providers, particularly those offering consistent information and emotional support played a critical role in helping patients feel seen and supported. The study highlights the need for more holistic, person-centered approaches to care that address not only the physical but also the psychosocial dimensions of life with an anal fistula.

## Introduction

Living with an anal fistula is often stressful and can be physically and emotionally challenging. Difficulties range from short-term challenges lasting weeks to those persisting for years or even being lifelong. Some fistulas heal spontaneously without surgery while others can be highly complex, requiring multiple surgical procedures over many years. Surgical approaches vary and the number of treatment strategies reflects the complexity of the condition [1]. New methods are arising, but there is no single treatment that can help all fistula patients.

Despite the diversity of symptoms in patients with anal fistula, research into patient experience is sparse, with few quantitative and qualitative studies published. Questionnaire studies on quality-of-life show a reduction in perceived quality-of-life [2–4]. This absence is particularly notable given how chronic and stigmatized conditions, even those less publicly visible, can exert a profound impact on how individuals construct and protect their sense of self in daily life. One survey examined various treatment modalities, noting that some of the more aggressive options carry the risk of fecal incontinence that significantly diminishes quality-of-life [3]. Another study revealed that patients with Crohns disease desire more information on the disease and the pros-and-cons of various surgical strategies so they can take part in intervention decision-making [5]. One survey covering a wide range of anorectal disorders measured the impact of benign anorectal conditions such as constipation and incontinence on quality-of-life. Quality-of-life was lower in affected individuals than in age-matched individuals without anorectal issues [6]. Another qualitative study showed that Crohns patients with a fistula where the future outcome was uncertain were severely affected physically and emotionally, and that this had a great impact on their quality-of-life. This included missed work opportunities, poorer personal relationships, and limited tolerance to mistreatment [2]. Lee *et al* stressed the patient’s need for information on the risks and benefits of fistula treatment [7]. Although anal fistula is a localized and often hidden condition, it shares important psychosocial features with other chronic illnesses. As seen in research on patients with cancer and similar long-term conditions, a loss of bodily control and persistent uncertainty can deeply affect how individuals perceive their agency and sense of self [8].

A study by Iqbal *et al* using semi-structured interviews showed the condition to have an impact far beyond just the physical symptoms [4]. Our current knowledge about the quality-of-life of patients with a fistula not related to Crohns disease remains limited, and there is a specific need for qualitative studies on other forms of fistula if we are to understand the patient’s situation, needs, and preferences and by so doing improve their care. Furthermore, studies that explore not just functional outcomes but how patients experience illness in relation to identity, stigma, and social participation are particularly lacking. Although anal fistula is a localized and frequently invisible condition, its psychosocial burden mirrors those seen in other stigmatized illnesses. Research on conditions such as cancer and depression has shown how individuals must renegotiate their sense of self in the face of disrupted bodily norms, social withdrawal, and persistent uncertainty [8,9]. Similarly, patients with endometriosis report symptom invalidation and frustration with care structures that fail to recognize the full scope of their experience [10]. These findings suggest a broader relevance of identity work, stigma, and social silence in shaping illness experience, and point to a gap in qualitative exploration of such processes in the context of anal fistula.

The aim of this study was to explore how patients with an anal fistula experience life, focusing on psychological aspects and the impact on daily living. In this study, we understand identity as a dynamic and socially influenced process, shaped by how individuals interpret their bodies, roles, and relationships over time [11,12]. Our analysis contributes a conceptual framework grounded in theories of illness identity and the disruption of everyday life within the context of a chronic condition. [11,13,14] This theoretical lens guides the study’s interpretation and aims to inform both future research and clinical practice.

## Materials and Methods

### Study design

An explorative qualitative study was conducted using semi structured interviews and qualitative content analysis [15]. The interviews were carried out by the first author, an M.D and PhD-student who is a surgeon specifically interested in proctology though not treating this group of patients clinically. This role supported a balance of insider knowledge and professional distance. The first author designed the interview procedure after discussions with two of the other authors. The interview contained open and follow-up questions. The design was tested in two interviews, followed by discussions within the team. No changes to the design were found necessary.

### Participants and recruitment

Participants were recruited from a University Hospital and a Regional hospital. These two populations differ regarding number of immigrants and educational levels. Inclusion criteria were: [1] age 18 years or older, and [2] a clinical diagnosis of anal fistula confirmed by a colorectal surgeon. Participants were recruited at the outpatient clinic by their surgeon in a consecutive order. All patients with fistulas undergoing or expected to undergo multiple treatment sessions were asked to participate during the study period. Patients with fistulas that did not require current treatment were not approached. Patients with mild fistula symptoms were not included, meaning those with simple, low fistulas that were well-drained, asymptomatic, or resolved after a single minor intervention. Such patients typically did not require ongoing surgical management or follow-up and were therefore outside the scope of this study. The study focused on individuals with fistulas of varying complexity who were undergoing active or repeated surgical treatment. This ensured that participants had direct, current experience of the physical, emotional, and social challenges associated with active disease and treatment.

Furthermore, exclusion criteria were: [1] inability to understand written or verbal study information sufficiently to provide informed consent, [2] cognitive impairment or severe psychiatric condition that could interfere with participation in an interview, and [3] inability to communicate in the language in which interviews were conducted (Swedish). All eligible patients who met these criteria and were undergoing active treatment were invited to participate. No patients meeting inclusion criteria were excluded for language or cognitive reasons during recruitment.

Shortly after being recruited, participants were contacted by the first author by telephone and given the opportunity to ask questions. After this, informed consent was obtained prior to inclusion. To assure anonymity, participants were assigned an anonymous code as soon as they were included in the interview study.

Seventeen people agreed to participate in the study. Seven men and eight women between 20 and 76-years-of-age were included (two patients did not answer the telephone call). The participants had different ethnic backgrounds, a variety of symptoms and varying duration of their fistula, as well as differences in fistula complexity. All were undergoing treatment during the study period and the inclusion process was halted when data saturation was obtained. Though selection was random, patients with very mild fistula symptoms were not included as they did not require surgical treatment. Inclusion criteria were 18 years and older and a diagnosis of anal fistula. Exclusion criteria were inability to understand written or verbal information; cognitive impairment; and inability to speak the language the interviews were held in.

### Data collection

Initial contact was made by telephone, during which a brief introduction to the study was repeated. If the participant agreed to take part, a convenient time for the interview was arranged. Informed consent was confirmed and documented prior to each interview. All participants were reminded that they could withdraw from the study at any time, during or after the interview.

All interviews, except one, were conducted over the phone. One participant chose to meet in person, and the interview took place in a hospital conference room. While participants were informed of the interviewer’s research role, they did not know her personally and she was not involved in their clinical care. All interviews were digitally recorded and transcribed verbatim by a medical secretary. The interviews took place February 10^th^ through May 26^th^ 2022, with one additional interview conducted on December 20^th^, 2022. The interviews lasted between 19 and 73 minutes. Participants were given the chance to read the transcribed version of their interview. No notes were taken during the conversation. However, a memo was written shortly after each interview to capture the interview context and key observations prior to analysis.

### Data Analysis

The data were analyzed using qualitative content analysis following the approach of Graneheim [15]. To begin with, meaning units relevant to the research aim were identified from the transcription of each interview. Second, these meaning units, outlining narratives, were condensed and labelled with codes on a low empirical abstraction level. The codes were then grouped into subcategories and higher-order categories through iterative comparison and abstraction. Three authors participated in coding and interpretation: the interviewer, a clinical expert in fistula care, and a qualitative researcher with no clinical background. This diversity in professional perspective supported analytic rigor and reflexivity. Data saturation was continually discussed between the authors during the study period. Data collection halted when no new categories or themes emerged, suggesting thematic saturation. Microsoft Excel 2024 (Microsoft Corp, Redmond, WA, USA) was used to structure and analyze data. Diverse interpretive perspectives across the research team helped ensure a balanced analysis. The team regularly reflected on how their assumptions and professional backgrounds might influence interpretations, striving to center the participants’ voices and lived experiences throughout. The study is reported according to the Consolidated Criteria for Reporting Qualitative Research Recommendations (COREQ) [16].

### Ethical considerations

The study was approved by the Ethics Committee in Sweden 2021–05047. All participants gave written informed consent. The papers with informed consent were sent to the informants by post and returned prior to the interview. The study followed the principles of the Helsinki Declaration and prioritized participant confidentiality and autonomy.

## Results

### Participant Characteristics

The final sample included 15 participants (8 women and 7 men) between 20 and 76 years of age, all of whom were undergoing active treatment for a cryptoglandular anal fistula at the time of the interview. Participants represented a range of fistula complexities and treatment stages. Symptom duration ranged from a few months to more than ten years. See Table 1 below.

**Table 1:** Participant characteristics.

Participant	Sex	Age	Duration of fistula
1	Male	50	Recent diagnosis
2	Female	25	7 years
3	Male	29	2 years
4	Female	76	Recent diagnosis
5	Male	59	10 years
6	Female	23	2 years
7	Female	43	3 years
8	Female	37	4 years
9	Male	53	18 months
10	Male	40	Recent diagnosis
11	Male	27	2 years
12	Female	53	6 years
13	Female	67	2 years
14	Female	20	1 year
15	Male	63	20 months

All participants were living in Sweden and represented diverse ethnic and educational backgrounds. The diversity of symptom duration, age, and treatment experiences allowed for a broad exploration of psychosocial and identity-related aspects of living with an anal fistula.

### Categories and subcategories

Interview analysis revealed the main category “Identity” in patients with an anal fistula. We further identified three categories “Identity-shaping mechanisms”: Knowledge uncertainty; Expectations and Experience; and Quality-of-life. These three categories were further divided into twelve subcategories providing deeper insights into each one of the identified mechanisms. “Identity-shaping mechanisms” refer to the social, emotional and informational forces that influence and mold how individuals understand and position themselves in the context of illness, e.g., their beliefs, and sense of self over time. See Table 2 for and overview of the categories.

**Table 2:** Overview of the analysis process.

Main-category	Category	Subcategory	Quote	
**Identity**	***1 Knowledge Uncertainty***	*a) Uncertainty about time waiting for surgery*	I have now had surgery twice. Apparently, several interventions are needed. The doctor estimated two to three operations six to eight weeks apart. I also have to visit a nurse every 14 days to clean the sore, so there’s a lot of misery that needs to come out.“	
			“I wish I had received more information about how long everything would take. I understand I have a complicated fistula, but I would like more exact information about how much treatment remains. If that’s not possible, I would at least like to hear the different possible scenarios. The same applies to how much time I’m likely to be on the waiting list for surgery.”	
			“Yes, I’ve reported the long queues—I’ve been critical about this. They don’t seem to prioritize patients like us since it’s not considered an emergency at the time, but it can become one.”	
		*b) Uncertainty about the disease*	“But they don’t appear to know much about it. I don’t either—I haven’t had much help. I know very little about fistulas. I’d heard of them before, but not the problems they can lead to.”	
		*c) Dealing with knowledge uncertainty through social contacts*	“I’d like to meet others with a fistula, to swap experiences. It would be interesting to hear how others manage—such as whether they’ve started to cycle again, and how it’s going. Things like that, everyday stuff. How do they deal with discharge? How do they handle their relationships and marriage? I want to know how people live with this, from day to day”	
	***2 Expectations & Experience***	*a) Diffuse start*	“It all started with intense pain on the right side, which I initially thought was ovulation. When it continued, I called the gynae clinic and got an emergency appointment with the gynecologist. They referred me to the ER, so I went. A surgeon saw me, along with a doctor and someone else, but no one could explain what was wrong. They thought it was a diverticulum or something. I went home completely exhausted. I lay in bed, repeatedly waking up due to the pain. I called the GP in the morning. My doctor told me, ‘I can’t help you—you must go back to the emergency department.’”	
			“It was autumn 2018 or 2019, and I had had pain around the tailbone for some time. I saw a chiropractor and said something wasn’t right. Eventually, I felt a lump while showering and asked a colleague at the healthcare center to look at it. It was all quite vague, but we ordered an MRI that showed a large abscess and an internal fistula.”	
		*c) Fear of not getting better*	“I’m afraid of becoming incontinent, but I try not to dwell on it. I shut it out of my everyday thoughts, but the fear is there, lurking beneath the surface.”	
			“I fear it will never go away—that I’ll have this string-like fistula for the rest of my life. I try to live as normally as possible, of course, but it feels like a lot of things have come to an end. I’ve lost so much already. It’s hard to accept.”	
		*d) Fear of privacy: Openness about the condition*	“All my friends know, but not all my colleagues—just the ones I’m close to. My boss arranged a special toilet with a bidet shower for me. Without it, I wouldn’t have been able to keep working.”	
			“I’ve been open with my boss from the start, so she understands. My schedule is adjusted if I get a date for surgery, they move me or change my shift, and I always have the day off afterwards. Most of my colleagues know, especially the ones I’m closest to. And my close friends and family know too. It’s not something I talk about—it’s just like having any other disease.”	
			“Only my daughters know. It feels too private to share with others. My friends know I’ve been to the hospital and had surgery, but I’ve only said it was something with my intestines. They maybe suspect more, but I’ve never told them why.”	
	***3 Quality of Life***	*a) Hygiene and secretion*	“It’s been really hard. I haven’t even thought about travelling or going out for a coffee. Even shopping for groceries is a challenge. I shower, prepare myself, and cover my underwear. When I get home, I repeat the whole process, shower again, change panty liners, and check that nothing has leaked onto my clothing. Eventually, I stopped going out unless I absolutely had to.”	
			“It definitely stops me from doing things—being in public places, going to concerts, dinners, parties where I can’t be sure there’s a toilet nearby. It’s mainly the hygiene part that holds me back. But otherwise, I feel that most things can be solved.”	
		*b) Intimacy*	“Things weren’t perfect before, but now feeling unclean and disgusting makes everything worse. My sex life has definitely suffered, and it’s affected our relationship.”	
			“After surgery, the area feels sensitive and unfresh. It absolutely affects my ability to be intimate with my wife.”	
		*c) Social interaction and relationships*	“Go out to restaurants or hang out somewhere with friends, things like that. When I have it I know I must take care of it, so I stay at home because I’m afraid that if I’m out a little longer something can happen with the patch and I’ll bleed a lot on my clothes which will be very uncomfortable.”	
			“You never feel fresh because of discharge, and then you have to change your underwear and wash everything immediately. I don’t really want to meet people. You become more withdrawn—like being a hermit.”	“
		*d) Pain*	“It’s not only pain and fatigue, but also the fact that I can’t lift anything. I can carry my dog—he’s only 3 kg—but I cannot lift weights or a grocery bag. If I lift or bend too much, I bleed heavily from the backside and the pain becomes intense. If I push myself too much, I end up bedridden for days.”	
		*e*) *A wish for a normal life*	“I try to stay positive and believe things will improve. I live as normally as I can—being outdoors, working when I’m well enough. You just try to keep living an ordinary life.”	
			“It’s very difficult to care for my horse or go riding. Planning things with the family is hard. We want to travel and swim with my daughter, but that’s not possible right now. My husband wants to go abroad, to Thailand, but everything has to wait until this is resolved. And it’s taking a long time.”	

Table 2 is illustrating a brief overview showing the main category “Identity”, the three “Identity-shaping mechanisms” categories, and the subcategories.

### Identity

Participants described how anal fistula impacted their sense of identity, both in subtle daily moments and in broader questions of self-definition. Their narratives reflected tensions between who they were before the illness and who they were becoming as they adapted to chronic symptoms. It was hard to deal with uncertainty: how long will I have symptoms – will it be months, years, or will I never be free of my fistula? Fear of incontinence or having a stoma was described, as well as the conflict of living as usual on one hand while feeling that nothing is truly normal on the other. Sitting on a chair or in the car is painful making it difficult to concentrate, affecting both work and social life. One participant described:

*“It’s quite difficult. It’s hard to sit because I’ve just had surgery, I’m sore and it’s extra sensitive. It’s been going on for so long now that I’ve got used to it. You cannot be yourself.”* (Interview 4, female 76 years)

Many experienced a disruption in how they saw themselves at work, in relationships, and in their bodies. A recurring theme was the difficulty of maintaining a coherent sense of self when the illness was painful, private, and unpredictable. The main category “Identity” is further described and illustrated by the three “Identity-shaping mechanisms” and their subcategories:

#### 1. Knowledge uncertainty.

The first category “Knowledge Uncertainty” comprised three subcategories: a) Uncertainty about time waiting for surgical care; b) Uncertainty about the condition; and c) Dealing with knowledge uncertainty through social contacts. “Knowledge uncertainty” refers to the state of being unsure or unclear about the nature of the illness, its treatment trajectory, and its possible outcomes. It encompasses doubts about beliefs, facts, and information. This lack of clarity shaped how participants approached their care and how confident they felt navigating the healthcare system.

*“What I must mention is the need for more information, and if information cannot be given then at least describe possible scenarios.”* (Interview 1, male 50 years)

Participants emphasized the importance of reliable, ongoing information. Many described frustrations with not knowing what to expect next, or how long the condition would last. They commented about the lack of awareness of living with a fistula in society leading to difficulty talking openly about their experiences or find meaningful support. The participants highlighted the importance of access to a contact nurse, someone consistent they could turn to for information and reassurance during a prolonged course of care. For many, uncertainty was not just medical, but also emotional and relational.

#### 2. Expectations and experiences.

The second category “Expectations and experiences” comprised four subcategories, all describing worries: a) Uncertainty about the illness and its care from the beginning, leading to worries about not receiving correct treatment; b) Worry about it becoming a protracted process; c) Worry about things not getting better; and d) Worry about their condition being revealed instead of remaining private. Worry, in turn, leads to uncertainty regarding expectations, i.e., insight into possible outcomes, realistic view of how things may unfold, and what to expect from the healthcare system. Worry also affects experiences that encompass perceived encounters and events in the individual’s life. In this context it was the worry about not knowing what to do due to having diffuse symptoms in the beginning that were not recognized. This was exemplified by how long it took to come to the right clinic to receive correct surgical care. See Table 2 for an overview of this category with additional quotes. The participants often described a slow and fragmented care pathway, which contributed to feelings of helplessness and frustration. Many worried that they were not receiving the right treatment, or that the process would never lead to recovery.

One participant expressed

*“This has been going on for so long you think the situation has come to a standstill, but on second thought it hasn’t. It’s just slow and annoying.* “(Interview 9, male 40 years)

Participants engaged in what Charmaz (1995) describes as anticipatory identity work—the imaginative projection of feared future selves, such as incontinence or needing a stoma—shaping how they made sense of current suffering and future loss of control [11]. These imagined outcomes functioned as threats to personal dignity and social belonging, reinforcing their anxiety and shaping how they approached treatment. The stoma, although uncommon in fistula treatment, emerged in narratives as a powerful metaphor for loss of control and disruption of bodily boundaries. Even when medically unlikely, it was described as “worse than the fistula,” suggesting that fear was not based solely on clinical risk but on what the condition symbolized for participants’ self-image [17].

*“I am worried that it will never go away, that I will have this fistula and string for the rest of my life. I try to live as good a life as possible, but there are certain things I love in life I have lost such as swimming and working out.”* (Interview 13, female 67 years old)

Participants also spoke about the emotional burden of potentially disclosing their condition to others. The final identified subcategory was fear of their condition being revealed and losing privacy. Most participants felt they could communicate effectively with their closest family members and with their employer, but they were reluctant to disclose their condition to others. Some participants had told their friends, but many had chosen not to because their friends knew nothing of the condition, and they considered it to be too private. Depending on the kind of work, making necessary adaptations to their job was crucial to being able to manage their fistula effectively and continue to work.

*“All my friends know, but not all my colleagues - only the ones I’m close to. My boss arranged a bidet in one of the toilets that I have access to, otherwise it would not have been possible to work at all.”* (Interview 3, male 29 years old)

#### 3. Quality-of-life.

The third category, Quality-of-life, comprised five subcategories: a) Hygiene and secretion; b) Intimacy; c) Social interaction and relationships; d) Pain; and e) The wish for a normal life.

The main limitation imposed by having an anal fistula was the issue of hygiene, which was perceived to impact every aspect of life, often causing them to stay at home. It impacted on the participants’ sense of control and social confidence. Many described a constant fear of leakage, the need to change clothes, and the and the anxiety of people noticing. These concerns often led to reduced participation in public or social settings. For many, the ongoing need to think ahead, stay clean, and avoid awkward moments became exhausting over time.

Although not taken up spontaneously, intimacy emerged as a sensitive and embodied concern when participants reflected on their relationships during the interviews. Participants described it as a major barrier to forming new relationships and confirmed the impact it had on existing ones. Participants saw it as a major obstacle when seeking an intimate relationship, the fistula imposed a sense of bodily defectiveness that complicated sexual closeness and desire. These self-protective narratives reflect what Charmaz (1995) refers to as discursive self-management, in which individuals manage stigma and vulnerability by reshaping how they present their illness within relationships [11]. This process illustrates how illness identity is negotiated in relational contexts, shaped by concerns of stigma and rejection [13,18,19]. One young woman explained:

*“There is less desire for intimacy because you have pain and feel dirty down there. My partner understands but even so, it is difficult.”* (Interview 15, woman 20 years)

This distancing was not only physical, but also narrative, a means of reclaiming control over a body that had become uncertain, painful, and socially risky.

The category “Quality-of-life” further comprised subcategories of pain. Pain was described as constant or recurring yet often normalized. Several participants said they had learned to live with it, even as it affected their concentration, energy, and emotional resilience.

*“I feel it on and off every day. The combination of pain and the feeling. I have learned to live with the pain, I feel it has become like a part of me.”* (Interview 13, female 67 years)

The anal fistula had an impact on the participants’ social life, their behavior, and relationships with other people. They experienced shame, had less interaction in relationships, and hesitated to be the one to take the initiative. Many weren’t sure how much to share with others and had to decide when to stay private and when to ask for support.

The final subcategory outlined was “Wish for a normal life”, reflected participants’ efforts to reclaim continuity and coherence in their lives interrupted by the unpredictability and stigma of the fistula. Rather than seeking transformation or adjustment, participants longed to return to what was previously taken for granted: spontaneity, bodily trust, and participation in ordinary family life [14]. This longing can in line with Williams (2000) be understood as a form of “normalization work”, and a response to the way chronic illness can disrupt a person’s daily routines, future plans, and sense of stability [14].

*“I can never plan things for my family, I want to travel and swim with my daughter, but that isn’t possible. My husband wants to go to Thailand and be abroad more, but I feel everything must wait a bit until this problem is solved and that is taking a long time.”* (Interview 8, female 37 years)

These hopes for returning to normality were not only goals but served as coping strategies, ways for participants to maintain a sense of identity and personal continuity despite the challenges of their condition [17,20].

These findings, taken together, show how participants’ experiences of living with anal fistula were shaped by uncertainty, bodily discomfort, and emotional strain. Across all three categories, Knowledge Uncertainty, Expectations and Experiences, and Quality-of-Life, they described how the condition affected how they saw themselves and how they related to others. Physical symptoms such as pain, leakage, and fear of incontinence often lead to feelings of shame, guilt, and a strong desire to hide their condition. The private and often invisible nature of the fistula contributed to stigma, while unclear information and slow treatment processes added to participants’ emotional fatigue. This resonates with findings in other under-recognized conditions such as endometriosis, where patients experienced symptom invalidation that exacerbated emotional distress and undermined their confidence in care [10].

Participants worked to preserve a sense of normality and self-worth, even as the fistula challenged their routines, relationships, and self-image. Their stories show how living with anal fistula involved ongoing efforts to maintain identity, privacy, and dignity in the face of an unpredictable and socially sensitive illness.

**Fig 1:**
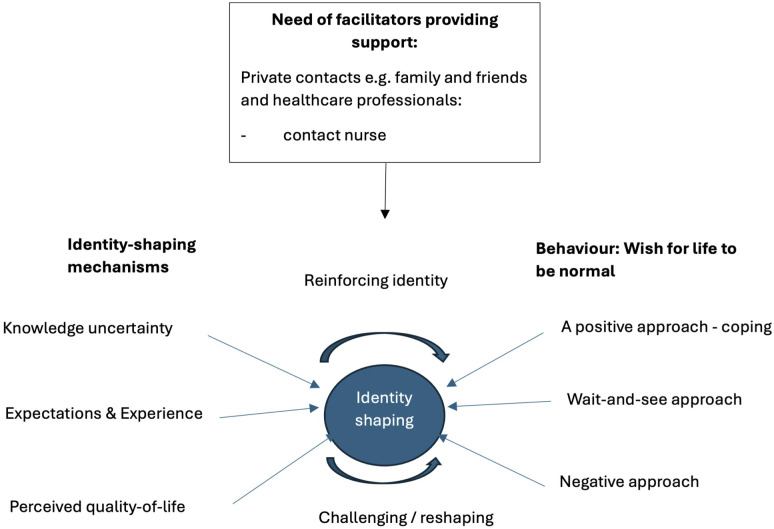
Identity-shaping mechanisms and response: key mechanisms that shaped identity for participants: knowledge uncertainty about their condition; expectations and experience; and lower quality-of-life. These factors collectively contribute to deterioration in self-image and how individuals with a fistula cope with time by interaction between physical health and identity.

Based on participants’ narratives, we developed a conceptual framework that identifies three identity-shaping mechanisms: Knowledge Uncertainty, Expectations and Experiences, and Quality-of-Life, as outlined in Fig 1. These categories were grounded in the data and reflect how participants negotiated their sense of self and social presence in the face of chronic symptoms, invisibility, and stigma. Each mechanism describes a set of experiences and emotional responses that challenged or reinforced how individuals viewed themselves, physically, socially, and emotionally.

## Discussion

This study explored how individuals living with an anal fistula experienced its impact on their psychological well-being, daily life, and sense of identity. Conflicting information or lack of adequate knowledge may lead to an identity crisis. Expectations and experiences play a crucial role in shaping how individuals regard themselves. See Fig 1 above. Positive experiences that align with expectations may reinforce an identity, while negative experiences may challenge or reshape it [21]. This reflects what Charmaz describes as the reconstruction of self in chronic illness, where identity is continually revised in response to bodily changes, uncertainty, and altered social roles. Understanding these connections can help address and manage the patients’ worries, that is psychological burdens associated with living with an anal fistula more effectively [11]. Personal examples, advice, and help in dealing with the stigma may reduce the patient’s fears and worries, improving their quality of life. This aligns with evidence that stigma can reduce patient engagement and prolong suffering [22]. Ensuring continuity of care and clear, compassionate communication could reduce the psychosocial burden of this condition. As such, our findings point to the need for a more stigma-aware and patient-centered approach to care.

Every participant longed for a normal life, being free of their fistula, and this had an impact on their behavior. We observed that participants approached their situation in various ways: some were positive and actively coped with their lives, while others adopted a passive stance, waiting to see what happens. Some took on a negative outlook, feeling disillusioned and sometimes engaging in negative behavior. These responses can be seen as different coping positions along a continuum of resilience, and was often closely linked to participants’ ability to maintain a coherent identity over time [23]. This aligns with research on cancer patients, where loss of bodily control and fragmented communication with healthcare professionals were found to shape disruptions in identity and agency. Over time, patients worked to regain a sense of coherence through dialogic and emotionally supportive clinical interactions [8].

Identity-shaping had a deep impact on the lives of the participants. A similar phenomenon was described for patients with Irritable Bowel Disease, where the diagnosis have a profound effect on family life, social life, and work; as well as the feeling of being controlled by the disease [24]. The participants in this study were forced to adapt their lives because of the fistula, finding ways to deal with the new situation. The disease directed their lives creating a new dimension of personal sacrifice but also a new insight into health and life. This aligns with Bury’s (1982) concept of chronic illness as a disruption to the expected flow of life, what he called a break in biographical continuity, requiring individuals to reinterpret their personal narratives. This experience has also been described in studies on how diabetes type 1, asthma, kidney failure and chronic heart failure effects self-image [11, 13, 25].

The term identity has been further explored by understanding “health identity” and “illness identity” which examine how people make sense of who they are in relation to illness. This aligns with Pelters’ (2024) framing of health and illness identity as dynamic constructs shaped by personal meaning, social interaction, and bodily experience. Health and illness are regarded as constructs and can be understood from questions like “Who are you with regard to health and illness?” or “How do you deal with health/illness?” [12]. How patients regard themselves changes with time as the condition is often ongoing, sometimes lasting several years. These constructs help frame the fluid and evolving nature of identity as participants adapt to a condition that may last for years and carries emotional and social meaning.

The three identity-shaping mechanisms presented here capture how patients navigated disruptions in knowledge, expectations, and everyday life, often without consciously framing it as identity work. Changes slowly develop as the individual adapts to the new way of life with their fistula, this is not always obvious to patients themselves. Yet their narratives reflect a deep engagement with questions of control, normalcy, and how to remain “themselves” despite prolonged uncertainty.

Matini *et al* studied how patients adapt life to inflammatory bowel disease, this patient group can also suffer fistula issues. Patients sought new definitions of what is normal in their lives just like the participants in this study [12,26]. This can be understood as a form of “normalization work” [20], where individuals strive to maintain or restore everyday routines and social relationships while managing a condition that threatens those very things.

A surprising finding was the fear of having a stoma even though this is an uncommon outcome of fistula disease. Many participants gave this answer to the question “What are you the most afraid of?” This fear appeared to reflect not only clinical concern, but a symbolic loss of bodily control and social acceptability, what Frank has described as the narrative weight where individuals imagine and emotionally respond to possible futures shaped by illness. None of the participants had accepted the condition as chronic, even those who had had it for years [27].

This study demonstrates that identity is not just disrupted by illness, but actively rebuilt through everyday struggles to stay visible, dignified, and connected. Narrative processes of making sense of illness over time, especially under unpredictable and socially charged conditions, can be seen as relational work. Research on cancer patients similarly illustrates how collective meaning-making with peers, family, and healthcare professionals can shape the experience of agency and illness identity [28]. Taken together, our findings align with recent qualitative work on other stigmatized or chronic conditions, reinforcing the importance of socially attuned care, identity-sensitive communication, and relational continuity in sustaining well-being across diverse health contexts [10,29].

### Limitations and suggestions for future research

While this study offers important insights into the lived experience of individuals with anal fistula, certain limitations should be acknowledged. Identity is shaped by social and cultural factors, and this may have influenced our findings. The hospital catchment areas included in this study are as diverse as can be in Sweden, but treatment strategies are probably similar. However, language and literacy constraints introduced selection bias since interviews were conducted in Swedish, and exclusion criteria required the ability to understand study information and provide consent. Although no patient was excluded because they requested an interpreter, non-Swedish speakers and those with limited literacy may therefore be under-represented. Future studies should include multilingual materials and interpreter support to ensure a broader representation of patient experiences.

Moreover, treatment strategies and cultural norms may differ across countries and healthcare systems. Our findings are therefore contextually situated, and the transferability to other healthcare systems should be considered with caution. Studies at an international level including patients from different geographical regions, sociocultural settings and varying healthcare systems could provide richer and comparative insights into both shared and context-specific experiences of living with an anal fistula. Exploring how cultural norms shape willingness to disclose intimate health concerns and to seek support would further enhance understanding of the social dimensions of this condition.

Additionally, our conceptual framework of identity-shaping mechanisms, Knowledge Uncertainty, Expectations and Experiences, and Quality-of-Life, was developed inductively based on participant narratives. Future research could build on or refine, this framework by applying it in other contexts such as in countries with different healthcare structures, cultural norms or in comparison with other stigmatized chronic conditions. Additionally, most participants were in active treatment. Including individuals in remission or long-term recovery could offer further insight into how identity evolves over time.

## Conclusion

The impact of anal fistula is individual and the patient’s response to the condition is shaped by physical symptoms, emotional stress, and social concerns. This study shows that these experiences are deeply connected to identity, and we propose a conceptual framework consisting of three identity-shaping mechanisms: Knowledge Uncertainty, Expectations and Experiences, and Quality-of-Life. These mechanisms help illuminate how patients attempt to preserve self-understanding and dignity in the face of an unpredictable and often stigmatized condition. Support from family, friends, and health professionals was described as essential for coping. We identified the need for enhanced emotional support, especially given the long duration and complexity of treatment, which require continuity of care. A designated contact nurse, someone who provides consistent communication, reassurance, and follow-up throughout the treatment journey, was seen as especially important. Participants felt that such a role could reduce uncertainty, foster trust, and make the condition feel more manageable. Similar challenges have been documented in other long-term conditions, where person-centered philosophies often fall short in actual service delivery. Patients value continuity and relational care but frequently encounter fragmented systems that make this difficult to achieve [29]. Clinicians must improve the timing, clarity and delivery of information given to the patient, especially in the immediate postoperative period when patients often feel vulnerable and uncertain. In addition, opportunities for peer support, whether through patient groups or online platforms, may provide shared understanding and reduce isolation. Iqbal et al. (2022) have similarly emphasized the value of accessible and ongoing support for this group.

This study underlines the need for a more holistic approach to fistula management that goes beyond physical treatment. Addressing the emotional and social aspects, such as reducing stigma and providing psychological support, is crucial to improving overall wellbeing. Education and counselling aimed at improving knowledge and alleviating uncertainty and fear are essential components in helping patients regain control over their lives. Moreover, tailored interventions that focus on enhancing quality-of-life and identity reconstruction, while taking into account the psychosocial burdens involved, can help patients regain control over their lives and feel better supported throughout treatment. Living with an anal fistula is not only a medical condition but also a deeply personal and social experience that challenges patients’ sense of identity, dignity, and connection. Our findings call for clinical care that recognizes this complexity, not only through better treatment options, but also by fostering relational continuity, emotional validation, and clear communication. By centering the patient’s lived experience, healthcare professionals can help restore a sense of control and self-worth in a context too often marked by invisibility and silence. In doing so, care for anal fistula can move beyond the biomedical to embrace the full person behind the diagnosis.
